# Knowledge gaps among South African healthcare providers regarding the prevention of neonatal *group B streptococcal* disease

**DOI:** 10.1371/journal.pone.0205157

**Published:** 2018-10-05

**Authors:** Caris A. Price, Lionel Green-Thompson, Vijay G. Mammen, Shabir A. Madhi, Sanjay G. Lala, Ziyaad Dangor

**Affiliations:** 1 Department of Paediatrics & Child Health, Faculty of Health Sciences, University of the Witwatersrand, Johannesburg, South Africa; 2 Office of Teaching and Learning, Faculty of Health Sciences, University of the Witwatersrand, Johannesburg, South Africa; 3 Medical Research Council: Respiratory and Meningeal Pathogens Research Unit, Faculty of Health Sciences, University of the Witwatersrand, Johannesburg, South Africa; 4 Department of Science and Technology/National Research Foundation: Vaccine Preventable Diseases Research Chair, Faculty of Health Sciences, University of the Witwatersrand, Johannesburg, South Africa; 5 Perinatal HIV Research Unit, Faculty of Health Sciences, University of the Witwatersrand, Johannesburg, South Africa; LSTM, UNITED KINGDOM

## Abstract

**Objective:**

To evaluate obstetric healthcare provider knowledge regarding the prevention of *group B streptococcal* disease in South African infants.

**Methods:**

Questionnaires exploring knowledge, attitudes and beliefs around *group B streptococcal* prevention were administered to consenting doctors and maternity nurses in a tertiary academic hospital. Qualitative assessments (focus groups) were undertaken with junior doctors and nurses.

**Results:**

238 participants completed the questionnaire: 150 (63.0%) doctors and 88 (37.0%) nurses. Overall, 22.7% of participants correctly identified the risk-based prevention protocol recommended at this hospital. Most doctors (68.0%) and nurses (94.3%) could not correctly list a single risk factor. A third of doctors did not know the correct antibiotic protocols, and most (80.0%) did not know the recommended timing of antibiotics in relation to delivery. Focus group discussions highlighted the lack of knowledge, awareness and effective implementation of protocols regarding disease prevention.

**Conclusions:**

Our study highlighted knowledge gaps on the risk-based prevention strategy in a setting which has consistently reported among the highest incidence of invasive *group B streptococcal* disease globally. In these settings, education and prioritization of the risk-based intrapartum antibiotic strategy is warranted, but an alternative vaccine-based strategy may prove more effective in preventing invasive *group B streptococcal* disease in the long-term.

## Introduction

In neonates and young infants, *group B streptococcus* (GBS) is a leading cause of sepsis and meningitis globally [1, 2], with the highest incidence reported in Eastern and Southern Africa [3]. Intravenous intrapartum antibiotic prophylaxis (IAP) has reduced the burden of early-onset GBS disease (EOD) in the United States of America (USA) by almost 90% over two decades [4]. Pregnant women with recto-vaginal GBS colonization between 35–37 weeks of gestational age are administered IAP at least four hours prior to delivery (universal screening). An alternate but less effective strategy to screening-based prophylaxis is to provide IAP to women with risk factors associated with subsequent neonatal invasive GBS disease [5].

Despite the success of EOD prevention in the USA, universal screening cannot be implemented in most low and middle income countries for various logistical reasons (lack of health care infrastructure and health care personnel) [6]. A recent review assessing global IAP implementation practices found that in countries with an IAP policy, 42% used clinical risk factors rather than universal screening [7]. Coverage for clinical risk factor-based screening, however, was heterogeneous with a median coverage of only 29% (range 10–50%) [7]. Until such time as alternative strategies such as maternal vaccination are available, it is essential that existing practices be optimized for the prevention of GBS disease.

In this setting, the burden of early-onset neonatal sepsis is estimated as 35.6 per 1000 live births, and 10% of cases are associated with bacteraemia, with GBS being the most commonly identified pathogen [8]. We have previously reported a high (and largely unchanged) incidence of EOD (1.41; 95% CI: 1.28–1.55) [3, 9]. This is most likely attributable to the poor implementation of the risk-based strategy: only 26% of pregnant women with risk factors actually receive IAP timeously [10]. Therefore, healthcare providers (HCPs) knowledge, attitudes and practices regarding GBS risk-based prevention are important. The aim of the present study was to evaluate current knowledge and healthcare practices in preventing invasive GBS disease among obstetric HCPs.

## Materials and methods

In the latter half of 2015, we conducted a prospective study in which we administered questionnaires to obstetric HCPs in a large tertiary referral hospital in Johannesburg, South Africa. Although South Africa is classified by the World Bank as an upper-middle income country, it is nuanced by its history of racial segregation with a legacy of vast inequality, including vast health disparities between different racial groups. This hospital serves a mostly Black-African population of low-income earners (<US$2 per day) with a high unemployment rate (53%) [11, 12]. In this region, pregnant women deliver either at this hospital (approximately 22,000 births annually) or at the midwife-obstetric units (approximately 10,000 births annually) [13]. The maternal GBS colonization rate is estimated between 21–33% [10, 14, 15].

At this hospital, the first interaction between HCPs and pregnant women in labour happens in the *Admissions* room, which is managed by 2–4 intern doctors and 6–8 nurses; this care is overseen by an obstetrics resident (registrar) or an attending (consultant) obstetrician. Thereafter, pregnant women are transferred to either the *First Stage* area or the *Labour Ward* depending on whether they are in the latent or active phases of labour respectively. In these areas, they are managed by midwives and assistant nursing staff with resident supervision.

The standard-of-care at this hospital for the prevention of GBS disease is the risk-based rather than universal screening strategy. Women with the following risk factors: (1) spontaneous preterm labour (<37 weeks), (2) membranes ruptured ≥18hours, (3) intrapartum pyrexia ≥ 38°C, (4) a previously affected baby with EOD or (5) urine, vaginal or rectal cultures positive for GBS should receive intravenous *ampicillin* during labour. In penicillin-allergic women, erythromycin or vancomycin is recommended. Pregnant women should be screened for risk factors in all three “labour” areas by the attending HCP.

The study was conducted in two parts: (1) a questionnaire-based evaluation on specific areas of knowledge of the above protocol-based GBS prevention guidelines and (2) focus group discussions around attitudes and perceptions towards GBS prevention. The specific sections are further defined below.

The questionnaire included questions on the perceived importance, transmission, risk factors and antibiotic use for GBS prevention (S1 Appendix). The questionnaire was developed and administered by the investigators (CP, SGL and ZD) and only protocol-defined risk factors were accepted as “correct” answers. A spot assessment of intern doctors, nurses, medical officers, residents and attending obstetricians working in the Obstetric Department was undertaken. We also included interns who worked in the Obstetric Department in the preceding 20 months; for the reason that in South Africa, interns complete a two year internship that includes a mandatory four month rotation in the Obstetric Department. The nursing staff comprised of registered midwives (professional nurses who have completed additional postgraduate training in midwifery), midwifery students, professional nurses (nurses who have completed a four year degree in nursing science), enrolled nurses (nurses who have completed a three year diploma), and enrolled nursing auxiliaries (nurses who have completed a basic one-year nursing course and who work under the direct supervision of professional or enrolled nurses). Written informed consent was obtained from participants and the study was approved by the University of Witwatersrand Human Research Ethics Committee (HREC number: M150616).

Two focus groups were held following completion of the questionnaires. The focus group discussions were chaired by an experienced qualitative researcher (LGT) with CP and ZD also present. The purpose of the focus group was to explore, in an open discussion, local site-specific issues that contribute to poor execution of the IAP protocol. Following a blend of purposive and convenience sampling, participants were confidentially consented to participate in the focus group discussion irrespective of whether they completed the questionnaire. The focus group discussions were held in English as all participants were fluent in English. The discussions were conducted in a meeting room at the hospital during the course of the normal working day (permission was granted for their absence from the ward). Both meetings lasted approximately 30 minutes and occurred only once. After a brief introduction to the chair and the research goals, the focus groups began with broad questions, such as, “What is your perception of the importance of GBS infection within this hospital?” and “What is your perception about the effectiveness of the current strategies aimed at reducing GBS infection in this hospital?” The first group discussion was held with eight nurses (four senior midwives and four students training in advanced midwifery nursing), three of whom had completed the questionnaire. The second focus group comprised 12 interns (three of whom had completed the questionnaire). No participants refused involvement or dropped out. Focus group discussions were audio-recorded and accompanying field notes were taken. LGT used a content analysis framework to analyse and consolidate the raw data. No software was used in data management and feedback was not given to participants.

The quantitative analysis focused on assessing the degree of knowledge of GBS burden and prevention. We analyzed all participants as a single group but also analyzed groups separately on the *a priori* premise that knowledge would differ between these groups based on their years of training and experience: (i) interns, (ii) nurses, and (iii) medical officers, residents and attending obstetricians (who were regarded as senior doctors). Comparisons were made using the chi-squared or Fisher’s exact test for proportions and the Mann Whitney test for medians. Data was analysed using STATA version 13.1 (College Station, Texas, USA).

## Results

The questionnaire was completed by 238 participants: 121 (62.7%) of 193 employed interns, 29 (48.3%) of the 60 senior doctors and 88 (53.7%) of 164 nurses working in the Obstetric Department. Of the 150 doctors, 121 (80.7%) were interns, 3 (2.0%) medical officers, 16 (10.7%) residents, and 10 (6.6%) attending obstetricians. Of the 88 nurses, 19 (21.6%) were advanced midwives, 44 (50.0%) professional, 15 (17.0%) enrolled, and 10 (11.4%) auxiliary nurses.

There were no significant differences in findings between interns currently working in the Obstetric Department and those that had worked there in the preceding 20 months; therefore, interns were analyzed as a single group. For nurses, there were no significant differences based on professional qualification and experience, and therefore nurses were also analysed as a single group. Prior to administering the questionnaire, the investigators had determined that unanswered questions would be regarded as incorrect responses. A sensitivity analysis showed no significant differences if unanswered responses were excluded.

Using a 10-point Likert scale, most participants felt that GBS is a significant pathogen causing neonatal sepsis as well as a substantial public health problem; medians were high (≥8) across the groups (Table 1). Table 1 also shows the proportion of participants who correctly answered questions to the questionnaire (S1 Appendix).

**Table 1:** Responses to the questionnaire by health-care providers.

	Overall n = 238	Doctors n = 150	Nurses n = 88
**1. Group B Streptococcus (GBS) is an important cause of infection in newborns**	n = 232	n = 149	n = 83
Median Likert score (Interquartile range; IQR)	**10 (8–10)**	**10 (8–10)**	**10 (7–10)**
**2. In our setting, how important of a public health issue do you think GBS is**	n = 231	n = 150	n = 81
Median Likert score (IQR)	**9 (7–10)**	**8 (7–10)**	**10 (8–10)**
**3. What is the commonest way in which newborns become infected with GBS**			
Correct response	164 (68.9)	117 (78.0)	47 (53.4)
**4. GBS can be transmitted to newborns during delivery and up to three months after delivery.**			
True	208 (87.4)	135 (90.0)	73 (83.0)
**5. What percentage of pregnant women have Group B streptococcus as part of their genitourinary and gastrointestinal flora**			
Correct response	83 (34.9)	59 (39.3)	24 (27.3)
**6. List 3 risk factors in the mother likely to increase the chance of GBS disease in her newborn**			
Nil correct	185 (77.7)	102 (68.0)	83 (94.3)
One correct	37 (15.6)	35 (23.3)	2 (2.3)
Two correct	12 (5.0)	9 (6.0)	3 (3.4)
Three correct	4 (1.7)	4 (2.7)	0 (0)
**7. Which preventative strategy does this hospital practice to prevent the spread of GBS to newborns**			
Correct response	54 (22.7)	47 (31.3)	7 (8.0)
**8. Which antibiotic might you prescribe/administer to a woman in established labor who is at risk of passing GBS to her newborn**			
Correct response	131 (55.0)	94 (62.7)	37 (42.1)
**9. When in relation to the delivery should intrapartum antibiotics be used?**			
Correct response	40 (16.8)	30 (20.0)	10 (11.4)
**10. How important to you is the implementation of the GBS prevention protocol**	n = 223	n = 144	n = 79
Median Likert score (IQR)	**10 (8–10)**	**9 (8–10)**	**10 (10–10)**

Footnote: Median and interquartile ranges are reported for questions 1, 2 and 10. The number and percentage in parenthesis are reported for the remaining questions

**Fig 1:**
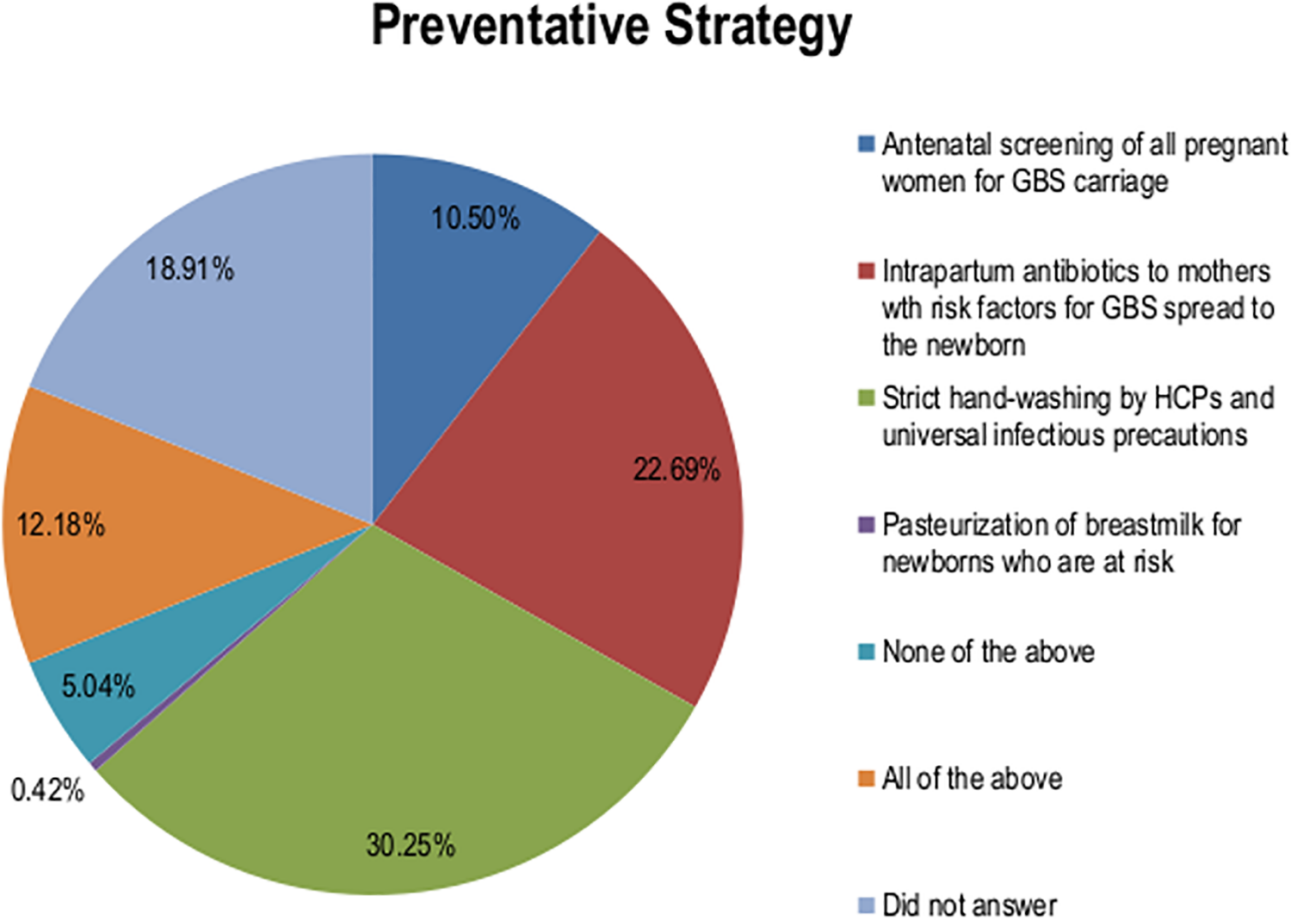
Healthcare provider (HCP) responses to the question regarding the *group B streptococcus* (GBS) preventative strategy.

Only 22.7% of participants correctly identified the risk-based screening approach to prevent EOD, and 77.7% of participants were unable to list a single risk factor for EOD (Fig 1 and Table 1). While 55.0% of participants correctly identified the appropriate antibiotic used, only 16.8% correctly noted the antibiotic should be given at least four hours before delivery to be effective (Table 1).

### Senior doctors compared to interns (S1 Table)

There was a significant difference (p = 0.001) regarding the correct listing of risk factors for GBS disease between senior doctors (55.2%) and interns (26.4%). As compared to interns, senior doctors were more likely to correctly identify the appropriate antibiotic to use (57.9% vs. 82.8%; p = 0.018).

### Senior doctors compared to nurses (S2 Table)

Senior doctors correctly listed risk factors more often than nurses (55.2 vs. 5.7%; p<0.001). Regarding the prevention of EOD, only 8.0% of nurses compared to 37.9% of senior doctors correctly identified the risk-based strategy practiced at this hospital (p<0.001).

### Focus group discussions

The findings of a lack of knowledge regarding EOD prevention were confirmed during focus group discussions. The group of nurses listed several issues, namely: the lack of information and knowledge about GBS as well as their unawareness of GBS-prevention protocols. Although participants experienced high pressure service load, this was not seen as the central issue in the lack of engagement with the GBS protocol. The nurses felt that feedback regarding the wellbeing of the neonate may sensitize them to the issue and improve their engagement with the protocol. The nurses further suggested that focused campaigns be conducted with media-based information as well as inclusion in their continuing education program. The reflections of participants suggested that greater attention be paid to the various components of the care of the neonate.

The intern focus group noted that their guide book contained information regarding GBS prevention but that this was not seen as a key activity in their daily work. They commented that their supervisors did not appear to prioritise the GBS protocol.

## Discussion

The main finding of this study was that HCPs working in this tertiary care setting with a high GBS disease burden had poor overall knowledge about risk-based GBS prevention protocols. This would result in an inability to identify pregnant women who may deliver an infant at increased risk of GBS disease and consequently missed IAP opportunities. These unexpected findings suggest failures of implementation of a risk-based strategy over many years. Although not necessarily generalizable to other low and middle income settings, HCP prioritization of risk-based GBS protocols probably feature much lower than obstetric emergencies such as eclampsia, foetal distress and emergency caesarean deliveries. The poor knowledge among the interns and nurses—i.e. the primary point-of-care HCPs, may be a consequence of their seniors’ limited ability to advocate the GBS prevention strategy possibly due to the high clinical workload.

The need for improvement in IAP using a risk-based strategy is also evident in high-resource settings. In New Zealand, risk factors were present in 55% of cases with EOD but only 31% received IAP [16]. Similarly, risk factors were present for 67% of EOD cases in the United Kingdom, of whom only 19% received IAP [17]. In these well-resourced settings, lack of knowledge of guidelines and/or the uncertainty regarding recommendations were cited as reasons for failure to institute IAP [16, 17]. Overall, these findings imply that in high, middle and low income countries, it is challenging to provide optimal IAP using the risk-based screening strategy.

Although universal microbiological screening of all pregnant women with the provision of IAP to GBS colonized women has been successful in high-income countries [4], this strategy is resource intensive and unlikely to be implementable in low and middle income country settings. The cost of routine recto-vaginal swabbing of pregnant women, laboratory infrastructure, the timeous relay of patient results and the administration of intravenous IAP at least four hours prior to delivery are some impediments to implementing the universal screening strategy in limited-resource settings. Although newer point of care molecular testing could address some of these impediments, its cost-effectiveness and logistical feasibility will need to be explored first.

Vaccinating pregnant women may be an alternative preventive strategy. A maternal GBS polysaccharide-protein conjugate vaccine has completed Phase-II trials [6]. It is hoped the vaccine will protect against not only EOD but also late-onset disease, the burden of which is unaffected by IAP [6]. Vaccination may be simpler and more cost-effective than the current IAP and screening strategies [18, 19]; it may also decrease GBS-associated preterm labour and stillbirth [20, 21]. Nonetheless, while a maternal vaccine is urgently warranted, IAP will be required in instances such as preterm labour and for un-vaccinated mothers. It is thus imperative that existing practices be optimised, especially in areas with high GBS disease burden and poorly implemented IAP strategies. In addition to creating awareness of GBS protocols, training HCPs to adequately implement the risk-based strategy is urgently warranted. Furthermore, GBS prevention guidelines should become a standard feature of the obstetric clerking notes.

Limitations of this study include that it was done on HCPs from a single tertiary centre only and therefore limits generalizability. The finding that HCPs believe GBS to be an important cause of neonatal sepsis and a public health priority may reflect a “questionnaire” bias. Although other risk factors for invasive GBS disease have been reported [22], in the questionnaire, only protocol-defined risk factors were accepted as correct.

In conclusion, knowledge of GBS disease prevention was suboptimal amongst HCPs in this setting, with knowledge gaps also existing in high income countries [16, 17]. An aggressive strategy is needed to address this unacceptable rate of early-onset GBS disease. Until maternal vaccination becomes an established strategy to prevent GBS disease, the reduction of GBS EOD is dependent on the successful provision of IAP. Studies evaluating the use of educational campaigns, checklists [23], or a pro forma that may improve the implementation rates of GBS prevention protocols are warranted.

## Supporting information

S1 Appendix Participant questionnaire. (DOCX)

S1 Table Comparison of responses to the questionnaire between senior doctors and interns. (DOCX)

S2 Table Comparison of responses to the questionnaire between senior doctors and nurses. (DOCX)
