# Supplementary material for: Knowledge gaps among South African healthcare providers regarding the prevention of neonatal group B streptococcal disease
Source: PLoS One. 2018 Oct 5;13(10):e0205157. doi: 10.1371/journal.pone.0205157 (PMC6173416; doi:10.1371/journal.pone.0205157)
Supplement: S1 Appendix — (DOCX) [file pone.0205157.s001.docx]

**S1 Appendix: Participant questionnaire**

**QUESTIONNAIRE**

**Please tick the block corresponding to your current job title.**

**Doctor:** Intern □ When did you do your Obstetrics block? mth/yr to mth/yr

Medical Officer □ Registrar □ Consultant □

**Nurse:** Enrolled nurse □ Professional Nurse □ Advanced Midwife □

1. *Group B Streptococcus (GBS) is an important cause of infection in newborns.*

| 1 | 2 | 3 | 4 | 5 | 6 | 7 | 8 | 9 | 10 |
| --- | --- | --- | --- | --- | --- | --- | --- | --- | --- |

Strongly Disagree Strongly Agree

1. *In our setting, how important of a public health issue do you think GBS is, in our setting?*

| 1 | 2 | 3 | 4 | 5 | 6 | 7 | 8 | 9 | 10 |
| --- | --- | --- | --- | --- | --- | --- | --- | --- | --- |

Not Important at all Extremely Important

1. *What is the commonest way in which newborns become infected with GBS?*

□ via health care worker

□ vertical (mother to newborn)

□ GBS-colonized breastmilk (i.e. the milk contains GBS)

1. *GBS can be transmitted to newborns during delivery and up to three months after delivery.*

□ True □ False

1. *What percentage of pregnant women have Group B streptococcus as part of their genitourinary and gastrointestinal flora (normal bacteria that live in the body and usually do not cause disease)?*

□ <10% □ 10-20% □ 20-30% □ >30%

1. *List 3 risk factors in the mother likely to increase the chance of GBS disease in her newborn?*

1.­­_______________________

2._______________________

3._______________________

1. *Which preventative strategy does this hospital practice to prevent the spread of GBS to newborns? Please choose the most correct answer.*

□ Antenatal screening of all pregnant women for GBS carriage

□ Intrapartum* antibiotics to mothers with risk factors for GBS spread to the newborn

□ Strict hand-washing by health care workers and universal infectious precautions during delivery

□ Pasteurization of breastmilk for newborns who are at risk

□ None of the above

□ All of the above

*Intrapartum = around the time of delivery

1. *Which antibiotic might you prescribe/administer to a woman in established labour who is at risk of passing GBS to her newborn? Please choose one only.*

□ IV Ampicillin

□ IV Cefazolin

□ Oral Amoxicillin

□ Oral Erythromycin

□ IV Cephalexin

1. *When in relation to the delivery should intrapartum antibiotics be used? Choose the most correct answer.*

□ Never, intrapartum antibiotics do not form part of the this hospitals preventative strategy.

□ At least 8 hours before delivery

□ At least 4 hours before delivery

□ At least 2 hours before delivery

□ At the time of delivery

□ Only at C-section

1. *How important to you is the implementation of the GBS prevention protocol?*

| 1 | 2 | 3 | 4 | 5 | 6 | 7 | 8 | 9 | 10 |
| --- | --- | --- | --- | --- | --- | --- | --- | --- | --- |

Not Important Very Important
