# Supplementary material for: Knowledge gaps among South African healthcare providers regarding the prevention of neonatal group B streptococcal disease
Source: PLoS One. 2018 Oct 5;13(10):e0205157. doi: 10.1371/journal.pone.0205157 (PMC6173416; doi:10.1371/journal.pone.0205157)
Supplement: S1 Table — (DOCX) [file pone.0205157.s002.docx]

**S1 Table:** Comparison of responses to the questionnaire between senior doctors and interns

|  | **Senior doctors n=29 (%)** | **Interns**  **n=121 (%)** | **p-value*** |
| --- | --- | --- | --- |
| **1. Group B Streptococcus (GBS) is an important cause of infection in newborns** | n=28 | n=121 |  |
| Median Likert score (Interquartile range; IQR) | 10 (8-10) | 10 (8-10) | 0.738 |
|  |  |  |  |
| **2. In our setting, how important of a public health issue do you think GBS is** |  |  |  |
| Median Likert score (IQR) | 9 (7-9) | 8 (7-10) | 0.692 |
|  |  |  |  |
| **3. What is the commonest way in which newborns become infected with GBS** |  |  |  |
| Correct response | 24 (82.8) | 93 (76.9) | 0.621 |
|  |  |  |  |
| **4. GBS can be transmitted to newborns during delivery and up to three months after delivery.** |  |  |  |
| True | 23 (79.3) | 112 (92.6) | **0.033** |
|  |  |  |  |
| **5. What percentage of pregnant women have Group B streptococcus as part of their genitourinary and gastrointestinal flora** |  |  |  |
| Correct response | 11 (37.9) | 48 (39.7) | 0.863 |
|  |  |  |  |
| **6. List 3 risk factors in the mother likely to increase the chance of GBS disease in her newborn** |  |  |  |
| Nil correct | 13 (44.8) | 89 (73.6) | **0.001** |
| One correct | 8 (27.6) | 27 (22.3) |  |
| Two correct | 5 (17.2) | 4 (3.3) |  |
| Three correct | 3 (10.4) | 1 (0.8) |  |
|  |  |  |  |
| **7. Which preventative strategy does this hospital practice to prevent the spread of GBS to newborns** |  |  |  |
| Correct response | 11 (37.9) | 36 (29.8) | 0.394 |
|  |  |  |  |
| **8. Which antibiotic might you prescribe/administer to a woman in established labor who is at risk of passing GBS to her newborn** |  |  |  |
| Correct response | 24 (82.8) | 70 (57.9) | **0.018** |
|  |  |  |  |
| **9. When in relation to the delivery should intrapartum antibiotics be used? Choose the most correct answer** |  |  |  |
| Correct response | 5 (17.2) | 25 (20.7) | 0.800 |
|  |  |  |  |
| **10. How important to you is the implementation of the GBS prevention protocol** | n=25 | n=119 |  |
| Median Likert score (IQR) | 10 (9-10) | 9 (8-10) | 0.091 |

*p-value calculated using the chi-squared or Mann Whitney test
