# Supplementary material for: Knowledge gaps among South African healthcare providers regarding the prevention of neonatal group B streptococcal disease
Source: PLoS One. 2018 Oct 5;13(10):e0205157. doi: 10.1371/journal.pone.0205157 (PMC6173416; doi:10.1371/journal.pone.0205157)
Supplement: S2 Table — (DOCX) [file pone.0205157.s003.docx]

**S2 Table:** Comparison of responses to the questionnaire between senior doctors and nurses

|  | **Senior doctors**  **n=29 (%)** | **Nurses**  **n=88 (%)** | **p-value*** |
| --- | --- | --- | --- |
| **1. Group B Streptococcus (GBS) is an important cause of infection in newborns** | n=28 | n=83 |  |
| Median Likert score (Interquartile range; IQR) | 10 (8-10) | 10 (7-10) | 0.465 |
|  |  |  |  |
| **2. In our setting, how important of a public health issue do you think GBS is** |  |  |  |
| Median Likert score (IQR) | 9 (7-9) | 10 (8-10) | **0.001** |
|  |  |  |  |
| **3. What is the commonest way in which newborns become infected with GBS** |  |  |  |
| Correct response | 24 (82.8) | 47 (53.4) | **0.005** |
|  |  |  |  |
| **4. GBS can be transmitted to newborns during delivery and up to three months after delivery.** |  |  |  |
| True | 23 (79.3) | 73 (83.0) | 0.657 |
|  |  |  |  |
| **5. What percentage of pregnant women have Group B streptococcus as part of their genitourinary and gastrointestinal flora** |  |  |  |
| Correct response | 11 (37.9) | 24 (27.3) | 0.277 |
|  |  |  |  |
| **6. List 3 risk factors in the mother likely to increase the chance of GBS disease in her newborn** |  |  |  |
| Nil correct | 13 (44.8) | 83 (94.3) | **<0.001** |
| One correct | 8 (27.6) | 2 (2.3) |  |
| Two correct | 5 (17.2) | 3 (3.4) |  |
| Three correct | 3 (10.4) | 0 (0) |  |
|  |  |  |  |
| **7. Which preventative strategy does this hospital practice to prevent the spread of GBS to newborns** |  |  |  |
| Correct response | 11 (37.9) | 7 (8.0) | **<0.001** |
|  |  |  |  |
| **8. Which antibiotic might you prescribe/administer to a woman in established labor who is at risk of passing GBS to her newborn** |  |  |  |
| Correct response | 24 (82.8) | 37 (42.0) | **<0.001** |
|  |  |  |  |
| **9. When in relation to the delivery should intrapartum antibiotics be used? Choose the most correct answer** |  |  |  |
| Correct response | 5 (17.2) | 10 (11.4) | 0.412 |
|  |  |  |  |
| **10. How important to you is the implementation of the GBS prevention protocol** | n=25 | n=79 |  |
| Median Likert score (IQR) | 10 (9-10) | 10 (10-10) | **0.009** |

*p-value calculated using the chi-squared or Mann Whitney test
